# Analysis of fungal microbiota diversity and potential pathogenic fungi in oral secretions and gut feces of captive giant pandas

**DOI:** 10.3389/fmicb.2025.1522289

**Published:** 2025-02-13

**Authors:** Xiaoping Ma, Xiangwen Zeng, Zhi Huang, Gen Li, Ruiguo Liu, Rongyan Luo, Xinni Li, Shanshan Ling, Chengdong Wang, Yu Gu

**Affiliations:** ^1^College of Veterinary Medicine, Sichuan Agricultural University, Chengdu, China; ^2^China Conservation and Research Center for the Giant Panda, Chengdu, China; ^3^College of Life Sciences, Sichuan Agricultural University, Chengdu, China

**Keywords:** giant panda, oral fungi, gut fungi, seasonality, opportunistic pathogens

## Abstract

**Background:**

Maintaining good oral and gut health is essential for the wellbeing of animals, and fungi are key components of the oral and gut microbiota. This study aims to explore the diversity and seasonal dynamics of oral and gut fungal communities in captive giant pandas, with a focus on their potential functional roles in health and digestion.

**Methods:**

In the study, we collected saliva and fecal samples from 60 captive giant pandas were collected in different seasons, oral and gut fungi were analyzed using internal transcribed spacer (ITS) amplicon sequencing. We used *α* and *β* diversity analyses to examine the differences in species diversity and composition among the different seasons. Furthermore, we validated the ITS amplicon sequencing results through fungal isolation and identification.

**Results:**

Analyses of *α* and *β* diversity revealed both the differences and similarities between the fungal communities in the oral and gut microbiomes of giant pandas. Ascomycota and Basidiomycota were predominant in both oral and gut groups, while the dominant genera in the four seasons were *Cutaneotrichosporon*, and *unidentified_Chaetothyriales_sp*. Additionally, *Cladosporium* and *Candida* were predominant in the oral and gut fungus, respectively, across all four seasons. Notably, fungal abundance and diversity in the oral microbiome were significantly higher than in the gut microbiome, a pattern observed throughout most seasons. Several potentially pathogenic fungi, such as *Fusarium*, *Candida* and *Aspergillus*, were detected in healthy giant pandas, with most showing increased abundance during winter. It is worth mentioning that we found a distinct bias in the functional communities of oral and gut fungi. The abundance of saprophytic fungi in the gut is relatively high, which may be related to their role in cellulose digestion.

**Conclusion:**

The abundance and diversity of fungal communities in the oral cavity and gut of giant pandas exhibit significant seasonal variations. While the oral cavity hosts a higher abundance and diversity of fungi, the species composition of fungal community composition is similar to that of the intestines. The majority of gut fungi are likely derived from the oral cavity or diet, the significant seasonal variation in gut fungal community structure further suggests that long-term resident fungi may not be present in the gut.

## 1 Introduction

The giant panda is a rare and endangered species endemic to China, and is also a symbol of global biodiversity conservation (Zeng et al., 2007). Giant pandas belong to the order Carnivora and possess a typical carnivorous gastrointestinal tract. Their genome lacks the enzymes required for digesting cellulose and hemicellulose. However, their long evolution has enabled giant pandas to develop a unique eating habit dominated by bamboo, eating for more than 14 h a day (Wei et al., 2012; Jin et al., 2021). Oral ecology plays a significant role in the health and well-being of giant pandas. The oral microflora is a complex ecosystem composed of bacteria, fungi, viruses, and other microorganisms. Suitable temperatures, humidity, pH, and complex structures in the oral cavity provide suitable growth environments for microorganisms. The human oral cavity has the second richest microflora after the gastrointestinal tract, with more than 700 species of bacteria and more than 100 species fungi. Fungi is an integral part of the oral microflora, *Candida* is the most commonly found fungus, followed by *Cladosporium*, various yeast, *Aureobasidium*, *Aspergillus*, *Fusarium,* and *Cryptococcus* (Ghannoum et al., 2010; Seed, 2014; Verma et al., 2018). The gut microbiota is considered a pivotal factor in regulating host health and has also been a focal point in investigating the fiber-digesting capabilities of giant pandas. Thus far, the majority of studies pertaining to the gut microbiota of giant pandas have predominantly focused on bacterial communities. However, fungi also play a crucial role in the gut ecosystem of animals. In addition to their direct impact on the host, such as fungal infections, fungi may also regulate changes in other microbial communities, thereby influencing host health through ecological competition (Mann et al., 2020; Zhu et al., 2021). Additionally, there are reports suggesting that fungi contribute to the digestion of cellulose and hemicellulose in the gut of giant pandas (Yang et al., 2018). The current research results indicate that the fungi present in the gut of giant pandas mainly belong to the phyla Ascomycota and Basidiomycota. At the genus level, predominant fungal genera include *Candida*, *Saccharomyces*, *Microidium*, *Pleospora*, *Myriangium*, *Trichosporon*, *Pythium*, *Fusarium*, *Aspergillus* and *Cryptococcus* (Yang et al., 2018; Zhan et al., 2020; Jin et al., 2021).

The delicate balance of microorganisms in the oral cavity is crucial for maintaining oral health and resisting the onslaught of external factors that can disrupt the equilibrium (Arweiler and Netuschil, 2016). When this balance is disrupted by certain factors, some normal microorganisms in the mouth are transformed into pathogenic microorganisms, ultimately resulting in a variety of related diseases (Avila et al., 2009). The dynamic changes in environmental conditions, dietary habits, and other factors associated with seasonal transitions can significantly impact the composition and diversity of oral fungal communities. Oral fungus within the oral cavity not only impact oral diseases such as dental caries, periodontal disease, and oral cancer but also affect diseases of the digestive and respiratory systems such as esophageal and pancreatic cancers, pneumonia, chronic obstructive pulmonary disease, and lung cancer (Gao et al., 2018; Dong et al., 2021; Zhong et al., 2021). In giant pandas, oral diseases are a prevalent issue, the prevalence of dental caries in giant pandas is high, and the prevalence rate of dental caries in captive giant pandas is significantly higher than that in wild giant pandas (Jin et al., 2012), seriously affecting their quality of life and health.

The normal gut microbiota of Giant Pandas constitutes a diverse community of microorganisms crucial for maintaining the balance and stability of their gut ecosystem. These microorganisms play pivotal roles in various aspects of Giant Panda health, including digestion and absorption of nutrients, metabolism, immune function, and development (Wei et al., 2015). Their intricate relationship with the health of Giant Pandas underscores their vital importance. The majority of pathogenic fungi found in the gastrointestinal tract are classified as opportunistic pathogens. Extensive research has demonstrated their pivotal role in the onset of conditions such as inflammatory bowel disease (IBD), irritable bowel syndrome (IBS), and antibiotic-associated diarrhea (AAD) (Krause et al., 2001; Cominelli, 2013; Sokol et al., 2017). Moreover, some studies have uncovered the presence of genes encoding cellulolytic enzymes within the gut fungi of giant pandas (Yang et al., 2018). These fungi may play a role in the digestion of cellulose in giant pandas by producing enzymes such as cellulases.

In this experiment, we conducted a comprehensive analysis of the oral and gut microflora of captive giant pandas across four seasons to elucidate the microbial ecology of the oral cavity and gut in giant pandas. Saliva samples and fecal samples from 15 captive giant pandas were collected in spring, summer, autumn, and winter, and the fungal communities were characterized using high-throughput sequencing. Our analysis revealed a remarkable shift in the composition and diversity of the oral and gut fungi between seasons, highlighting the influence of seasonal variations on the oral and gut fungal communities in giant pandas. Additionally, we conducted a comparative analysis of the fungal communities in the oral and intestinal microbiomes, elucidating both their similarities and differences in microbial composition. Our findings provide some insights into the dynamic interplay between the oral and gut fungi and environmental factors. We focused on the potential role of gut fungi in cellulose digestion in Giant Pandas and underscored the importance of understanding the oral and gut microbiota ecology for devising effective strategies to prevent and treat oral and gastrointestinal diseases in this endangered species.

## 2 Materials and methods

### 2.1 Sample collection

In this study, samples were collected from clinically healthy giant pandas (nine females and six males, aged 6–26) at The Dujiangyan Giant Panda base (Dujiangyan, China). None of the giant pandas had a record of antibiotic use for nearly 3 months. Giant pandas were fasted for 1 h before sample collection, and all samples were collected without anesthesia or restraint. Sterile swabs moistened with 0.9% NaCl solution or phosphate buffered saline (PBS) were used to collect samples from all tooth surfaces, which was repeated three times for each giant panda (Seed, 2014). Fresh fecal samples from giant pandas were collected within 30 min after defecation. The samples were carefully collected, excluding any parts in direct contact with the ground. A total of 15 giant pandas were collected in each season. The oral samples were numbered ISp1-15 (spring), ISu1-15 (summer), IAu1-15 (autumn), and IW1-15 (winter), with a total of 60 samples. The intestinal samples were labeled as ISpF1-15 (spring), ISuF1-15 (summer), IAuF1-15 (autumn), and IWF1-15 (winter), corresponding to the respective seasons. The sampling information of different season is shown in Table S1.

### 2.2 DNA extraction, PCR, and NGS sequencing

Genomic DNA extraction from oral swab samples was conducted using the cetyltrimethylammonium bromide (CTAB) method (Fulton et al., 1995), while fecal samples underwent genomic DNA extraction utilizing the DP712 magnetic bead-based extraction kit (TIANGEN, Peking, China) designed for soil and fecal genomic DNA, and the DNA quality of each sample was determined by 1% agarose gel electrophoresis. The ITS gene was amplified with the specific primers (ITS1: ITS5-1737F: 5’-GGAAGTAAAAGTCGTAACAAGG-3,’ ITS2-2043R: 5’-GCTGCGTTCTTCATCGATGC-3′) targeted conserved sequences found in fungi. The polymerase chain reaction (PCR) amplification system comprised a total volume of 30 μL, including 3 μL of each primer (6 μM), 15 μL of Phusion Master Mix (2×), 10 ng of template DNA, and ddH2O to attain the final volume. The PCR reaction protocol entailed pre-denaturation at 98°C for 1 min, followed by denaturation at 98°C for 10 s, annealing at 50°C for 30 s, extension at 72°C for 30 s, and a final extension at 72°C for 5 min. The amplified PCR products were then subjected to 2% agarose gel electrophoresis to confirm the expected product size and further purified using a QIAquick gel extraction kit (Qiagen, Hilden, Germany). Subsequently, sequencing libraries were generated and barcoded using a TruSeq® DNA PCR-Free Sample Preparation Kit (Illumina, San Diego, CA, USA). The quality of the library was evaluated using a Qubit@ 2.0 Fluorometer (Thermo Scientific, Waltham, MA, USA) and Agilent Bioanalyzer 2,100 system (Agilent Technologies, Santa Clara, CA, USA). Finally, sequencing was performed using a NovaSeq 6,000 sequencer with 250-bp paired-end reads (White et al., 2013; Ma et al., 2021b).

### 2.3 Data analysis

To ensure the accuracy of the data obtained from each sample, we removed the barcode and primer sequences from the raw reads. Next, we utilized FLASH (V1.2.7) to splice the reads, generating Raw Tags (Magoc and Salzberg, 2011). The resulting data underwent further filtering to obtain Clean Tags. To ensure high-quality and clean tags, we applied the QIIME (V1.9.1) quality filtering process with specific filtering conditions (Caporaso et al., 2010; Bokulich et al., 2013). The tags were compared with the reference database (Silva database) using the UCHIME algorithm (UCHIME^1^), and any detected chimera sequences were removed (Haas et al., 2011; Edgar, 2013). The resulting Effective Tags were clustered into OTUs using Uparse (v7.0.1001), annotated via Qiime (v1.9.1) with the Unit database (v8.2), aligned with MUSCLE (Edgar, 2004), and normalized for further analysis. All raw sequence data were uploaded to the National Center for Information Biotechnology Information Search^2^ under the registration number PRJNA715063 and PRJNA715079.

Rarefaction curves and Venn diagrams were generated using R software (v2.15.3). Alpha diversity indices (ACE and Shannon) were calculated with Qiime (v1.9.1), with significance assessed via the Wilcoxon rank-sum test (for pairwise comparisons) and the Kruskal-Wallis test (for multi-group comparisons). Beta diversity analysis was conducted based on Bray-Curtis and Binary Jaccard distances, computed in Qiime (v1.9.1) (Caporaso et al., 2010). Group differences in community composition were evaluated using PERMANOVA (adonis method), reporting R^2^ and *p*-values, while differences in data dispersion among groups were tested with betadisper, also reporting p-values. A UPGMA dendrogram was constructed based on Bray-Curtis distances to cluster samples. The LEfSe analysis threshold was set at LDA score ≥ 4 and *p* < 0.05 for significance. Functional guild annotation was performed using the FunGuild tool, predicting potential ecological roles (e.g., plant pathogens, saprotrophs) based on fungal taxonomic information.

### 2.4 Isolation and identification of fungus

Saliva and fecal samples were inoculated on Sabouraud Dextrose Agar (SDA) plates containing chloramphenicol and actidione and incubated at 25°C. Fungi were isolated and purified based on their morphological characteristics to obtain single colonies. Fungal DNA was then extracted using the Fast Fungal Genomic DNA Isolation Kit (Sangon Biotech, Shanghai, China). The fungal ITS region was amplified using universal primers ITS1 (5′-TCCGTAGGTGAACCTGCGG-3′) and ITS4 (5′-TCCTCCGCTTATTGATATGC-3′). The PCR system and program followed the method described by Ma et al. (2024b). The PCR products were sequenced by Sangon Biotech (Shanghai, China). The sequencing data were analyzed using NCBI BLAST, and a phylogenetic tree was constructed using MEGA5 to compare the sequencing data.

## 3 Results

### 3.1 Fungal sequencing date

Following filtering and splicing of the raw data, we generated a total of 3,765,835 high-quality tags for the oral groups and 3,877,186 high-quality tags for the gut groups. Sequencing depth was assessed by constructing rarefaction curves, which showed that the number of operational taxonomic units (OTUs) approached saturation, indicating sufficient sequencing coverage to capture most of the microbial diversity present in the samples (Supplementary Figure S1).

**Figure 1:**
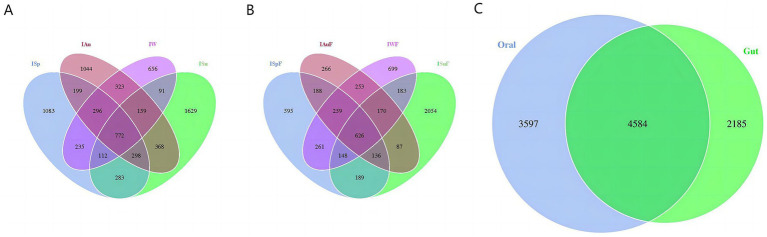
OTU distribution. The distribution of OTUs in oral group **(A)** and gut group **(B)** varied across different seasons. **(C)** The OTUs shared between oral and gut groups.

In the oral samples, we identified a total of 7,574 OTUs, with 772 OTUs shared among all four groups. Moreover, we detected 1,083, 1,629, 1,044, and 656 OTUs exclusively in the ISp, ISu, IAu, and IW groups, respectively (Figure 1A). In the gut samples, a total of 6,125 OTUs were identified. Among these, the ISpF, ISuF, IAuF, and IWF groups exhibited 2,402, 3,593, 1985 and 2,599 OTUs, respectively. Across all four seasons, a shared set of 626 OTUs was observed (Figure 1B).

Furthermore, we analyze the shared and unique OTUs between the oral and gut samples. There are 4,584 OTUs shared between the oral and gut groups, with 3,597 unique to oral group and 2,185 unique to gut group (Figure 1C). These findings indicate that our sequencing approach was capable of capturing the diversity of the microbiome across the different groups. A considerable portion of fungal species present in both the oral and intestinal environments are shared, and both exhibit their highest degree of uniqueness in fungal communities during summer.

### 3.2 Fungal diversity analysis

**Figure 2:**
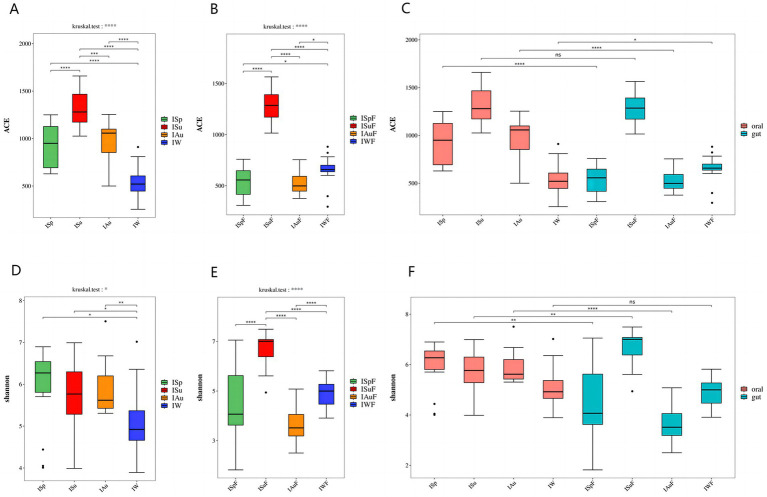
*α* diversity difference map: The ACE index revealed variations in fungal community richness across different seasons **(A)** oral group **(B)** gut group **(C)** oral group vs. gut group; The Shannon index revealed variations in fungal community diversity across different seasons **(D)** oral group **(E)** gut group **(F)** oral group vs. gut group. (*p* < 0.0001, ****; *p* < 0.001, ***; 0.001 < = *p* < = 0.01**; 0.01 < *p* < = 0.05, *; if *p* > 0.05, not marked).

The ACE (Abundance-based Coverage Estimator metric) index in both oral and gut samples indicates significant differences(*p* < 0.0001) among the four seasonal groups, with the summer group (ISu, ISuF) exhibiting the highest species richness compared to the spring (ISp, ISpF), autumn (IAu, IAuF), and winter (IW, IWF) groups, which also show significant differences(*p* < 0.001). In oral and gut samples, the species richness of the spring (ISp, ISpF), and autumn (IAu, IAuF) groups are similar, with no significant differences between the two groups. However, contrasting results are observed in the species richness of the winter group (IW, IWF) in oral and gut samples. In oral samples, the IW group exhibits lower richness compared to the ISp and IAu groups(*p* < 0.0001), while in gut samples, the IWF group shows higher richness compared to the ISpF and IAuF groups (*p* < 0.05) (Figures 2A,B). Comparing the ACE index between the oral and intestinal groups, the species richness in the oral samples is significantly higher than in the gut samples(*p* < 0.01). Specifically, in the summer group, there is no significant difference in the ACE index between the oral and gut samples. However, in the other three seasons, the ACE index of the oral group was significantly higher than that of the gut group, except in winter (*p* < 0.0001, *p* < 0.0001, *p* < 0.05) (Figure 2C and Supplementary Figure S2A).

Regarding the seasonal variation of oral fungal species abundance in captive giant pandas, the results indicate significant differences in the Shannon index among the four seasonal groups in both oral and gut samples (*p* < 0.05, *p* < 0.0001). In oral samples, the Shannon index across the four seasons from highest to lowest as ISp > ISu > IAu > IW. While no significant differences are observed between these three groups, the IW group displays significant differences compared to the other three groups (*p* < 0.05). In gut samples, the fungal species diversity is highest in the ISuF group, with significant differences observed compared to the other three groups (*p* < 0.0001). The fungal diversity between the ISpF and IAuF, IWF groups is not significant, but there is a significant difference between the IAuF and IWF groups (*p* < 0.0001) (Figures 2D,E). The Shannon index results indicate that the fungal community diversity is higher in the oral samples compared to the gut samples, with a significant difference observed between the two groups (Supplementary Figure S2B). Across all four seasons, the Shannon index of the oral samples exceeded that of the gut samples significantly, except during winter, where no notable difference was found, and in summer, where the oral samples had a significantly lower Shannon index than the gut samples (*p* < 0.01, *p* < 0.01, *p* < 0.0001) (Figure 2F). The ACE and Shannon index of oral and gut samples is shown in Supplementary Table S2.

### 3.3 Fungal community structure analysis

**Figure 3:**
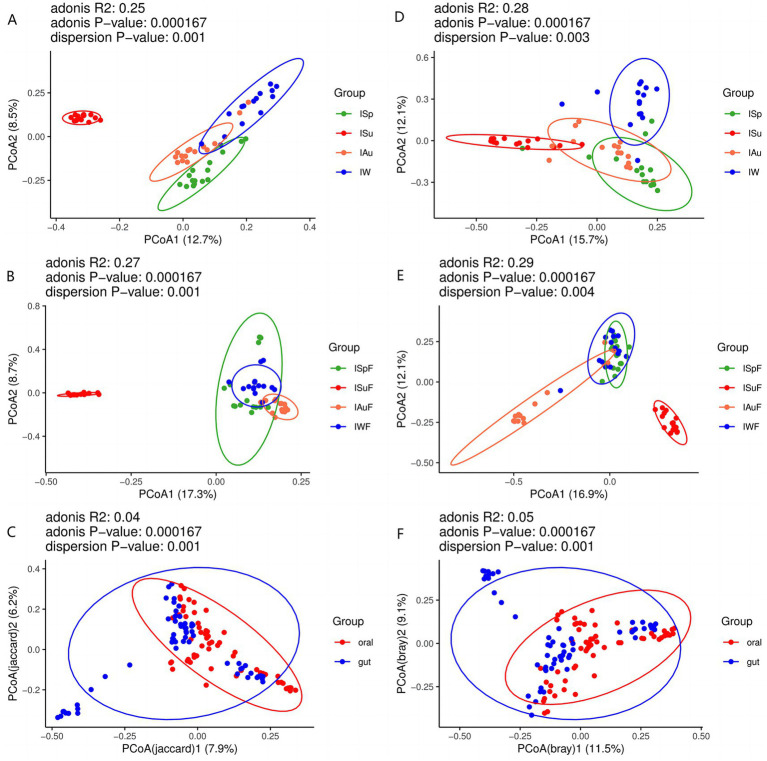
PCoA plot of oral samples based on Binary Jaccard **(A)** oral group across different seasons **(B)** gut group across different seasons **(C)** oral group vs. gut group; PCoA plot of oral samples based on Bray Curtis analysis **(D)** oral group across different seasons **(E)** gut group across different seasons **(F)** oral group vs. gut group.

To assess the differences in fungal community composition between oral and gut samples across the four seasons. we employed principal coordinate analysis (PCoA) based on Binary Jaccard and Bray Curtis analyses. In both oral and gut samples, the samples from the summer group (ISu, ISuF) are distantly distributed from the other three groups (ISp, ISpF, IAu, IAuF, IW, IWF), with no or minimal overlapping regions (Figures 3A,B,D,E). This suggests that in both the oral and gut environments, the fungal species composition and abundance in summer are significantly different from the other three seasons. In oral samples, there are varying degrees of overlapping regions among the ISp, IAu, and IW groups (Figures 3A,D), indicating noticeable differences in fungal community composition while also suggesting a certain level of similarity. In gut samples, there are overlapping regions among the ISpF, IAuF, and IWF groups, with the most prominent overlap observed between the ISpF and IWF groups (Figures 3B,E). This suggests that the fungal community structures in the gut samples of giant pandas are similar between spring and winter, while the fungal community in autumn samples exhibits greater uniqueness. These findings indicate that the oral and gut fungal community composition of giant pandas varies across different seasons, but also exhibits some degree of similarity. Specifically, the fungal community in the summer season displayed the highest degree of dissimilarity from those observed in the other seasons. The distribution of oral and gut samples exhibits considerable overlap, indicating a degree of similarity in the fungal community structure between the two environments. However, some gut samples are distantly distributed from oral samples, suggesting the presence of both similarities and differences in the fungal community structure between the oral and gut environments (Figures 3C,F).

Based on the Bray-Curtis distance, we conducted sample clustering analysis of oral and gut samples across all seasons. The clustering results of oral samples indicate that most samples from the spring and winter groups are clustered together in the same branch, except for samples IW2, ISp6, IW14, ISp13, and ISp6, which are not clustered with samples from the same season. In contrast, there are a few instances of inconsistent clustering among samples from the summer and autumn groups. For example, samples Au2, Au6, Au5, Au11, and Au12 are clustered within the summer group (Supplementary Figure S3A), suggesting a certain degree of similarity in the oral fungal communities between the summer and autumn seasons. From the clustering results of gut samples, it can be observed that most samples from the summer, autumn, and winter groups are clustered within their respective branches, with only a few exceptions such as ISuF6, IAuF4, IAuF13, IAuF11, IWF14, IWF4, and IWF2. However, the clustering results of samples from the spring group appear more scattered, dividing into three different clusters (Supplementary Figure S3B). This suggests that the structure of the intestinal fungal community in giant pandas during the spring season exhibits some similarity with the other three seasons. Clustering analysis of a total of 120 samples from both oral and intestinal sources reveals a scattered distribution pattern, indicating some complexity in the sample distribution (Supplementary Figure S3C). However, there is a tendency for samples from the same season, whether from the oral or intestinal sources, to cluster together.

Overall, the clustering results of samples from different seasons show differences in both oral and intestinal samples, indicating some variation in the fungal community composition. Samples from the same season exhibit similarities in fungal community composition but also demonstrate some differences.

### 3.4 Fungal community-composition analysis

**Figure 4:**
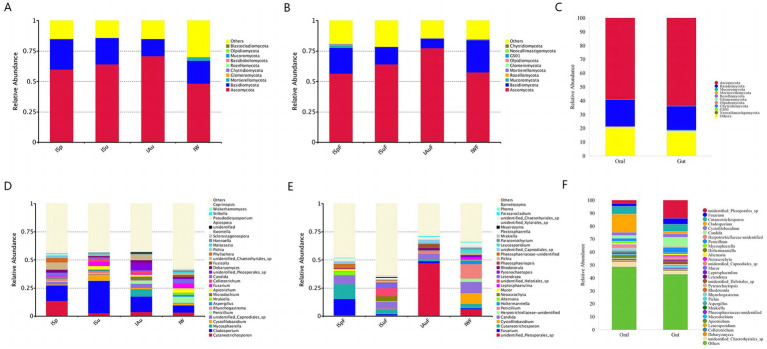
Microbial community bar plot showing average percentage of oral and gut fungus populations of giant pandas. Microbial community bar plot at the phylum level **(A)** oral group across different seasons **(B)** gut group across different seasons **(C)** oral group vs. gut group; Microbial community bar plot at the genus level **(D)** oral group across different seasons **(E)** gut group across different seasons **(F)** oral group vs. gut group.

The sequencing analysis revealed a total of 18 phyla, 72 classes, 202 orders, 510 families, and 1,338 genera in the oral samples. In the gut samples, a total of 18 phyla, 64 class, 189 orders, 475 families, and 1,184 genera were identified. At the phylum level, Ascomycota and Basidiomycota were the dominant taxa in both oral and gut groups, and the estimated cumulative abundance of these two phyla ranged from 60 to 80% (Figures 4A,B). It is worth noting that the top two fungal phyla were observed across all four seasonal groups in both oral and gut samples with only slight variations in their overall abundance (Figure 4C), indicating a high degree of similarity in fungal community composition between oral and intestinal samples across different seasons at the phylum level.

At the genus level, oral samples in the ISp group had the highest *Cutaneotrichosporon* content (13.84%), followed by *Cladosporium* (13.56%), *unidentified_Chaetothyriales_sp* (6.41%), *Phyllachora* (4.01%), and *Candida* (3.20%). *Cladosporium* (28.89%) had the highest content in the ISu group, followed by *Cystofilobasidium* (5.37%), *Fusarium* (3.86%), and *Apiotrichum* (3.11%). In the IAu group, *Cladosporium* (13.82%) is the predominant taxon, followed by *unidentified_Pleosporales_*sp. (6.69%), *Mycosphaerella* (6.49%), *unidentified_Chaetothyriales_sp*. (4.94%), *Cutaneotrichosporon* (3.78%), and *unidentified_Capnodiales_sp*. (3.64%). *Cladosporium* (6.27%) is the most abundant genus in the IW group, followed by *Penicillium* (5.36%), *Fusarium* (4.33%), *Apiotrichum* (4.13%), *Candida* (3.75%) and *Cutaneotrichosporon* (3.35%). Among the major fungal genera >1%, *Cladosporium*, *Cutaneotrichosporon*, and *unidentified_Chaetothyriales_sp* were found in all four seasonal groups, with average relative abundances of 15.64, 5.91, and 3.48%, respectively. The fungal community structure varied greatly between seasons and individuals, indicating a complex interplay between environmental factors and host factors (Figure 4D).

In the gut samples, the abundance at the genus level in the ISpF group is as follows: *Fusarium* (14.28%), *Cutaneotrichosporon* (13.13%), *Candida* (8.04%), *Alternaria* (2.93%), *unidentified_Chaetothyriales_sp* (2.08%) and *unidentified_Capnodiales_sp* (2.07%). In the ISuF group, the abundance of *unidentified_Helotiales_sp* (7.13%) is highest, followed by *Candida* (6.00%), *Letendraea* (4.60%), *Cladosporium* (4.44%), *Neoascochyta* (4.32%), *Cutaneotrichosporon* (4.31%), *Epicoleosporium* (2.94%). In the IAuF group, *unidentified_Pleosporales_sp* (47.36%) is he predominant taxon, followed by *Candida* (4.56%), *Phaeosphaeriopsis* (3.96%), *unidentified_Capnodiales_sp* (3.08%), *unidentified_Chaetothyriales_sp* (2.38%), *Penicillium* (2.37%). In the IWF group, the abundance at the genus level is as follows: *Penicillium* (12.88%), *Candida* (9.96%), *Cystofilobasidium* (9.75%), *unidentified_Pleosporales_sp* (6.33%), *Cutaneotrichosporon* (3.62%), *Holtermanniella* (3.19%), *Leucosporidium* (2.45%) and *Leptosphaerulina* (2.07%). The genera *Cutaneotrichosporon*, *Candida*, and *unidentified_Chaetothyriales_sp* are present in all four seasonal groups, with average relative abundances of 5.68, 7.14, and 1.72%, respectively. Significant differences in fungal community structure are observed among different seasons and individuals (Figure 4E).

Comparing the abundance at the genus level between oral and gut samples, significant differences in the fungal community structure of captive giant pandas are evident. However, genera such as *Cutaneotrichosporon*, *Candida*, *Cladosporium*, *Fusarium*, and *Cystofilobasidium* are distributed with relatively high abundance in both oral and gut environments (Figure 4F). These results suggest a certain degree of similarity between the fungal communities in the oral cavity and gut of giant pandas. This could be attributed to the possibility that some fungi in the gut originate from the oral cavity.

### 3.5 Fungi isolation and identification

We isolated and identified fungi from both oral and gut samples. In the oral samples, we identified 10 fungal species, while 7 species were identified in the gut samples. Among these, 5 species were found in both sample groups: *Cladosporium halotolerans*, *Cystofilobasidium infirmominiatum*, *Cutaneotrichosporon moniliiforme*, *Debaryomyces hansenii*, *Wickerhamomyces anomalus*. Additional fungi identified in the oral samples included *Apiospora arundinis*, *Aspergillus ochraceus*, *Beauveria bassiana*, *Cladosporium anthropophilum*, and *Pascua guehoae*. In the gut samples, *Aspergillus penicillioides* and *Fusarium equiseti* were identified. The majority of the isolated and identified fungal strains were dominant species either seasonally or throughout the year. The identification of these fungi supports the results of the community analysis. The evolutionary tree of the fungi is shown in Supplementary Figure S4. The NCBI accession numbers are presented in Table S3.

### 3.6 LEfSe analysis

In order to investigate the microbial community compositional differences among the four seasons, we employed the linear discriminant analysis effect size (LEfSe) approach, and the LDA scores are shown in Supplementary Figure S5. The results demonstrated significant differences among the four seasons in oral and gut sample (Supplementary Figures S5A,B). *Cutaneotrichosporon* and *Phyllachora* are representative and dominant in the ISp group; *Cladosporium*, *Cystofilobasidium*, and *Colletotrichum* in the ISu group; *Mycosphaerella*, *unidentified_Pleosporales_sp* and *Debaryomyces* in the IAu group; and *Penicillium*, *Apiotrichum*, and *Mortierella* in the IW group. In the gut samples, representative fungi in the ISpF group include *Cutaneotrichosporon*, *Fusarium*, and *Mucor*. While in the ISuF group, representative fungi include *unidentified_Helotiales_sp., Neoascochyta*, *Letendraea*, *Cladosporium*, *Epicoleosporium*, and *Pichia*. *Phaeosphaeriopsis* and *unidentified_Pleosporales_sp* are representative and dominant in the IAuF group. *Penicillium*, *Cystofilobasidium*, *Holtermanniella*, *Leucosporidium* in the IWF group. The LEfSe analysis results indicate that at the genus level, the representative and dominant taxon in the oral group is *Cladosporium*, while in the gut group, the representative taxon *unidentified_Pleosporales_sp* (Supplementary Figure S5C).

### 3.7 Analysis of potentially pathogenic fungi

**Figure 5:**
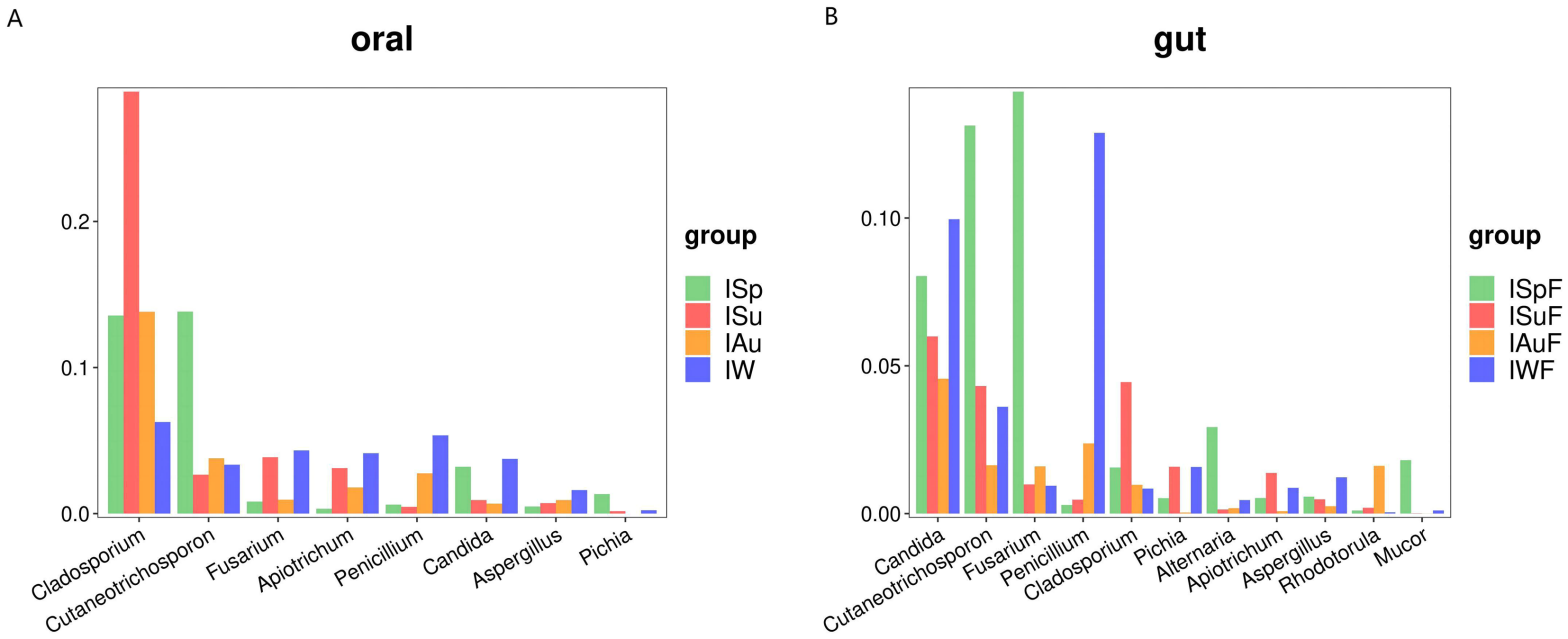
Comparative analysis of potential pathogenic fungal groups across different seasons. **(A)** Oral group, **(B)** gut group.

We conducted separate screenings of fungal communities in oral and gut samples from each season and combined the results with LEfSe analysis, focusing on taxa with abundances exceeding 1%, in an attempt to identify fungal communities that may be associated with potential pathogenic fungi. In our study, we found that the fungal communities present in both oral and gut samples indeed harbor some potential pathogenic fungi. In oral samples, potential pathogenic fungi with relatively high abundance include *Cladosporium*, *Cutaneotrichosporon*, *Fusarium*, *Apiotrichum*, *Penicillium*, *Candida*, *Aspergillus* and *Pichia* (Figure 5A). While in gut samples, *Candida* is the most abundant genus, followed by *Cutaneotrichosporon*, *Fusarium*, *Penicillium*, *Cladosporium*, *Pichia*, *Alternaria*, *Apiotrichum*, *Aspergillus*, *Rhodotorula* and *Mucor* (Figure 5B).

Both oral and gut samples exhibited notably high abundances of *Candida*, a common potential pathogenic fungus known to contribute to oral and gut diseases. Many members of the *Candida* are commonly found in the gut tract of animals, aligning with our findings. *Candida albicans*, the most common species within this genus, frequently infects humans and other animals, leading to conditions such as oral thrush and Candida enteritis. Additionally, we observed significant increases in the abundance of certain potential pathogenic fungi during certain seasons. For instance, the abundances of *Candida*, *Cutaneotrichosporon*, and *Fusarium* were notably elevated during the spring and winter seasons in oral and gut samples (Figures 5A,B). These fluctuations may be associated with environmental factors such as temperature and humidity, as well as host immune status and other variables.

### 3.8 Fungal function prediction

**Figure 6:**
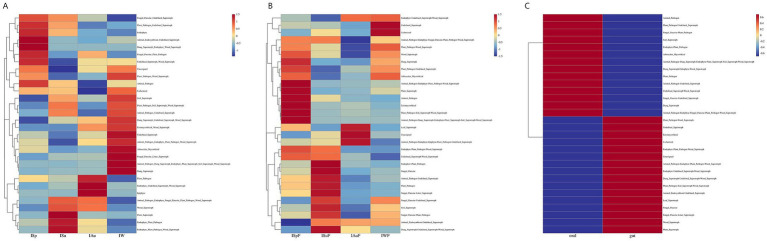
Heatmaps of fungal functional community distribution based on FUNGuild functional annotations in the oral and gut environments. **(A)** oral group across different seasons **(B)** gut group across different seasons **(C)** oral group vs. gut group.

In this study, we utilized the FunGuild tool to analyze fungal sequence data from oral and gut microbiome samples. This allowed us to explore their functional composition and ecological roles. In oral samples, the predicted fungal functions were assigned to 75 guilds, while in gut samples, they were assigned to 72 guilds. In samples from both oral and gut regions, fungal functions were assigned to 79 guilds. The top 30 categories were visualized. Across all four seasons, both oral and gut samples showed notably high abundance of saprotroph and plant-pathogen. However, the abundance of plant-pathogen was higher in oral samples compared to gut samples, while gut samples predominantly exhibited saprotroph abundance (Supplementary Figure S6). The clustering results demonstrated that the oral and gut fungal community’s functional composition was relatively stable within each seasonal group but significantly different between groups (Figure 6). These findings suggest that the oral and gut fungal community’s functional composition is subject to seasonal variation. Additionally, the heatmaps reveal differences in fungal communities between oral and gut samples. Oral samples exhibit a diverse range of fungal functions, including a higher abundance of saprotroph, numerous plant-pathogen, and some animal-pathogen. These differences may be closely related to the dietary habits and oral hygiene of giant pandas. In contrast, gut samples show significantly higher abundances of saprotroph, particularly wood-saprotroph. Overall, our study provides insight into the functional diversity and dynamics of the oral and gut fungal community, highlighting its potential role in oral and gut health and disease.

## 4 Discussion

As a rare and endangered species, the giant panda has been the subject of extensive research regarding its oral and gut microbiota. However, studies focusing specifically on the fungal communities in its oral and gut tracts remain scarce. Fungi constitute a minor component of the oral and gut microbial flora, yet their contribution to the overall biomass cannot be overlooked. Unlike bacteria, fungi have unique immunomodulatory properties, which can have divergent impacts on the host immune system through varied mechanisms (Niimi et al., 2010; Cui et al., 2013).

The results of this study reveal that the diversity and composition of oral and gut fungal communities in giant pandas vary significantly across seasons. The observed seasonal differences likely reflect shifts in environmental factors, diet, and host physiology. For instance, the higher fungal diversity in oral samples during spring and summer may be linked to increased dietary variety or changes in host behavior during these seasons. The findings align with previous studies suggesting that diet and seasonality can significantly impact microbial diversity in both the oral cavity and gut (Carey et al., 2013; Xue et al., 2015; Gao et al., 2018). Interestingly, the absence of significant differences in fungal diversity between oral and gut microbiomes during winter may indicate a convergence of environmental and dietary factors that affect both habitats similarly. This highlights the complex interplay between external influences, such as food availability, and internal host factors, such as immune responses, in shaping microbial communities. The lower fungal diversity observed in gut samples across all seasons is consistent with previous reports that most fungi in the gut are transient and do not colonize the gastrointestinal tract (Auchtung et al., 2018). Unlike bacteria, fungi often exhibit limited adaptation to gut-like conditions, and their abundance is influenced more directly by oral or dietary inputs. Seasonal differences in gut fungal diversity could therefore reflect the variation in dietary composition rather than stable colonization. The findings also support the notion that the oral microbiome is more dynamic than the gut microbiome. Factors such as diet, age, gender and hygiene are well-known to influence oral and microbial diversity (Anukam and Agbakoba, 2017; Adler et al., 2016; Liu et al., 2020). For example, the differences in oral fungal diversity observed here align with previous studies showing that die, such as the type of food consumed, can significantly alter microbial diversity (Adler et al., 2016). Zhang et al. (2018), reported that with the increasing age of giant pandas, the number of gut fungal OTUs also increased. However, no significant differences in fungal abundance were observed between different age groups (juveniles and cubs), and changes in fungal community composition showed no apparent correlation with age (Zhang et al., 2018). Strati et al. (2016), studied the effect of gender on the human gut fungal community and found that females had a greater number and diversity of fungal isolates compared to males, which may be associated with the regulation of sex hormones (Strati et al., 2016). By integrating these findings with prior research, this study highlights the intricate relationships between seasonality, diet, and microbial dynamics in different niches of the host. Future studies could explore the functional roles of these fungi in the health and ecology of giant pandas, shedding light on how fungal communities contribute to host fitness and adaptation in a changing environment.

The diversity of oral and gut fungi in giant pandas shows significant seasonal variation. Oral fungal diversity and abundance are lowest in winter, with peak abundance in summer and peak diversity in spring. In the gut, fungal richness and diversity are lowest in autumn and highest in summer. Overall, oral fungi exhibit higher diversity and abundance than gut fungi, except in summer and winter, where no significant differences are observed. Many factors may influence the changes in oral fungal diversity. Previous studies on oral microbes have shown that factors such as age, season, and diet can affect oral and gut microbial diversity (Carey et al., 2013; Xue et al., 2015; Gao et al., 2018). The community structure of oral microbial flora did not exhibit temporal stability. With increasing age, the *α* diversity of oral microbial flora decreases, whereas the *β* diversity increases (Anukam and Agbakoba, 2017; Liu et al., 2020). Additionally, the oral microbial flora also varies with different food. Adler et al. (2016) reported that the oral microbial diversity of cats fed dry food was higher than those fed wet food (Adler et al., 2016). While in the gut, most fungi are transient and do not routinely colonize the gastrointestinal tract. Auchtung et al. (2018), investigated fungal colonization in the gastrointestinal tracts of healthy adults and found that fungal abundance was very low across various diets. These fungi did not grow when cultured under gut-like conditions. Moreover, changes in oral hygiene or diet directly influenced the presence of two common fungi in fecal sample (Auchtung et al., 2018).

Ascomycota and Basidiomycota are the dominant fungal phyla in oral and gut groups, maintaining their dominance throughout all seasons. In oral group, the composition of fungal communities is relatively stable, with *Cutaneotrichosporon*, *Cladosporium*, and *unidentified_Chaetothyriales_*sp. being present in all seasons. Notably, *Cutaneotrichosporon* (15.64%) and *Cladosporium* (5.91%) remaining consistently high abundance throughout the year, although the abundance of other genera exhibited seasonal variation. The composition of oral fungal communities in giant pandas were similar to the human oral fungal communities reported by Ghannoum et al. (2010), where Ascomycetes and Basidiomycetes were dominant, *Candida*, *Cladosporium* and *Fusarium* were highly abundant in the oral microbial flora of both humans and giant pandas. In contrast, the gut fungal community demonstrated pronounced seasonal variations. Among them, *unidentified Pleosporales_sp* dominated the gut community in autumn and winter, with their abundance reaching nearly 50% in autumn but declining sharply in spring and summer. Additionally, certain gut fungi maintained relatively high abundance throughout the year, including *Cutaneotrichosporon* (5.68%), *Candida* (7.14%), and *unidentified Chaetothyriales sp.* (1.72%). Their consistent presence across all four seasons suggests a potential role in maintaining the stability of the gut environment. This differs somewhat from previous studies on the gut fungal community structure of giant pandas, as species such as *unidentified Pleosporales* and *unidentified Chaetothyriales* were rarely reported before (Yang et al., 2018). In our study, the multi-factorial nature of our sampling protocol may have contributed to the fluctuating fungal community dynamics. Giant pandas show seasonal preferences for different parts of bamboo plants (Hansen et al., 2010). In response to the seasonal growth patterns of bamboo, caretakers provide various types of bamboo to the pandas throughout the year. In addition, high temperatures, humidity, and frequent precipitation in the spring and summer may lead to a large number of microorganisms in the environment, thus affecting the fungal community in the oral cavity and gut, increasing its diversity. Temperature and humidity are usually lower in winter, which may inhibit the growth of microorganisms in the environment, and thus reduce the diversity of fungal communities in the oral cavity and gut. Notably, the structure of the oral fungal community exhibits little variation throughout the year, with *Cladosporium* and *Cutaneotrichosporon* consistently maintaining dominance. In contrast, the gut fungal community structure shows significant seasonal differences. *Unidentified_Pleosporales_sp* is the dominant fungi in autumn and winter, with its abundance approaching 50% of the gut fungal community in autumn. However, the abundance of *unidentified_Pleosporales_sp* is very low in spring and summer.

In the oral and gut microbiomes of giant pandas, we observed several potentially pathogenic fungi with high abundance, including *Cladosporium*, *Cutaneotrichosporon*, *Fusarium*, *Apiotrichum*, *Penicillium*, *Candida*, *Aspergillus*, *Pichia*, *Alternaria*, *Rhodotorula*, and *Mucor*. Notably, *Alternaria*, *Rhodotorula*, and *Mucor* were more abundant in the gut, although they were also present in the oral microbiome at lower abundances. Among these fungi, *Candida* is a common opportunistic pathogenic fungus found in the oral and gut microbiomes of giant pandas. It is present across all four seasons, with higher abundances observed in spring and winter. Various systemic or local factors can lead to excessive growth of *Candida* in oral mucosa, leading to the occurrence of oral candidiasis (OC), among which *Candida albicans* is the most common pathogen (Castillo-Martinez et al., 2018). In the gut, *Candida* can potentially cause various intestinal diseases like inflammatory bowel disease (IBD) (Sokol et al., 2017), and translocate to the bloodstream, causing life-threatening deep infections (Perez, 2019). The higher abundance of *Candida* in the oral cavities and gut of captive giant pandas, especially in spring and winter, may indicate an increased risk of OC or IBD in these animals. *Aspergillus*, which was abundant in oral samples collected during winter, is an opportunistic pathogen that usually causes infection after the body’s immunity is damaged or suppressed. Cho reported a case of locally aggressive palatal aspergillosis caused by *Aspergillus* infection in a patient with acute leukemia, with symptoms including fever and cellulitis on the roof of the mouth(Cho et al., 2010). The increase in *Aspergillus* content in the mouth of giant pandas in winter may increase the risk of oral *Aspergillus* infection. Furthermore, there are few reports of oral or bowel diseases caused solely by the other potentially pathogenic fungi identified. *Fusarium* can cause various infections, including superficial infections, localized invasive infections, and disseminated infections, with symptoms varying by the host’s immune status and the site of infection (Nucci and Anaissie, 2007). *Cladosporium* is known to be an opportunistic pathogen that can cause asthma and superficial and deep infections in humans, and has also been reported in skin and vaginal samples from healthy giant pandas (Ma et al., 2021a; Yue et al., 2021). *Apiotrichum* and *Cutaneotrichosporon* can cause systemic trichosporonosis, typically leading to superficial infections, though there are occasional reports of these fungi causing septicemia (Nath et al., 2018; Li et al., 2023). The majority of *Penicillium* species are harmless to humans, but some species can cause invasive infections in immunocompromised individuals (Lyratzopoulos et al., 2002). In addition, opportunistic pathogens such as *Alternaria*, *Rhodotorula* and *Mucor* found in oral and gut samples, were present at relatively lower abundances compared to other potential pathogenic fungi and primarily cause superficial infections (Tuon and Costa, 2008; Woudenberg et al., 2015; Baumgardner, 2019). It is important to note that the identification of these potentially pathogenic fungi in the oral fungal communities of captive giant pandas does not necessarily imply that these animals are suffering from fungal infections. Further research is needed to investigate the potential health implications of these findings and to identify the factors contributing to the observed differences in fungal abundance and diversity among seasons. Nonetheless, our study provides important insights into the oral fungal communities of giant pandas and highlights the need for continued monitoring and management of their health in captivity.

Currently, there is no consensus on the relationship between oral and gut fungal communities, particularly regarding whether oral fungi can migrate along the digestive tract and establish themselves as resident fungi in the gut. Previously, it was suggested that fungi in the oral cavity can enter the digestive tract with food and saliva. Some of these fungi may colonize the gastrointestinal mucosa and become resident fungi, while others are excreted with feces (Ott et al., 2008; Hamad et al., 2012; Hoffmann et al., 2013). However, recent studies increasingly suggest that there may be little to no resident fungi in the human gut. Most fungi present in the gut are likely transient and are ultimately excreted with feces. For instance, Auchtung et al. (2018), reported that fungi detected in gut and fecal samples were also present in the diet or oral cavity, with fungal colonization in the gut may be more indicative of disease occurrence (Auchtung et al., 2018). In our study, the abundance and diversity of fungal communities in the oral cavity were significantly higher than those in the gut. While the fungal community structures in the oral and gut environments showed significant differences, there were also notable similarities. Most fungi found in the gut were also present in the oral cavity, albeit at different abundances. Genera such as *Cutaneotrichosporon*, *Candida*, *Cladosporium*, *Fusarium*, and *Apiotrichum* were relatively abundant in both the oral and gut environments. These findings provide evidence that gut fungi may entirely originate from the oral cavity or diet, and the significant seasonal variations in the gut fungal community structure further suggest that there may not be long-term resident fungi in the gut. Additionally, this study used fresh fecal samples as a representation of gut fungal communities, which may lead to limitations and inaccuracies in our understanding.

It is worth mentioning that we found the abundance of saprotroph in the gut to be significantly higher than in the oral cavity, which may be related to cellulose digestion. These wood-associated saprotrophs primarily decompose cellulose and lignin in plant residues, suggesting these fungi in the gut microbiota may complement the host’s lack of enzymatic capacity for cellulose digestion, contributing to energy extraction from their fibrous diet. Among these saprotrophic fungi, *Aspergillus* has been extensively studied for its ability to produce cellulases, including endoglucanases, exoglucanases, and *β*-glucosidases, which degrade cellulose (Ali et al., 2015; Ma et al., 2024a). In the gut group of this study, a relatively high abundance of *Aspergillus* was also observed. This genus, or similar fungal taxa, may enhance the host’s digestive efficiency by synergistically breaking down plant fibers. In contrast, the functional groups of oral fungi were more diverse, including a high abundance of plant-pathogen and saprotroph, as well as some animal-pathogen. This diverse fungal guild in the oral cavity may be due to its direct contact with the external environment, making it more susceptible to environmental fungal influences.

## Data Availability

The datasets presented in this study can be found in online repositories. The names of the repository/repositories and accession number(s) can be found in the article/Supplementary material.

## Footnotes

^1^ http://www.drive5.com/usearch/manual/uchime_algo.html

^2^ https://www.ncbi.nlm.nih.gov/

## Supplementary material

SUPPLEMENTARY FIGURE S1 **(A)** Rarefaction curve of oral group. **(B)** Rarefaction curve of gut group. When the curve tends to be flat, the amount of sequenced data is reasonable.

SUPPLEMENTARY FIGURE S2 **(A)** Comparison of the ACE index between the oral and gut groups. **(B)** Comparison of the Shannon index between the oral and gut groups.

SUPPLEMENTARY FIGURE S3 UPGMA Clustering Tree Based on Bray Curtis. **(A)** Oral group, **(B)** gut group, **(C)** oral group vs gut group.

SUPPLEMENTARY FIGURE S4 Evolutionary relationships of taxa. The evolutionary history was inferred using the Neighbor-Joining method. **(A)** Oral group and **(B)** gut group.

SUPPLEMENTARY FIGURE S5 LEfSe analysis of fungal communities (LDA>4, *P*<0.05). LEfSe analysis of oral **(A)** and gut **(B)** fungal communities across different seasons. **(C)** LEfSe analysis of fungal communities between the oral and gut environments.

SUPPLEMENTARY FIGURE S6 Fungal functional category bar plot showing average percentage of FUNGuild functional annotation relative abundance in oral fungus. **(A)** Oral group across different seasons, **(B)** gut group across different seasons, and **(C)** oral group vs gut group.
