# Supplementary figures and images for: Analysis of fungal microbiota diversity and potential pathogenic fungi in oral secretions and gut feces of captive giant pandas
Source: Front Microbiol. 2025 Feb 13;16:1522289. doi: 10.3389/fmicb.2025.1522289 (PMC11881749; doi:10.3389/fmicb.2025.1522289)

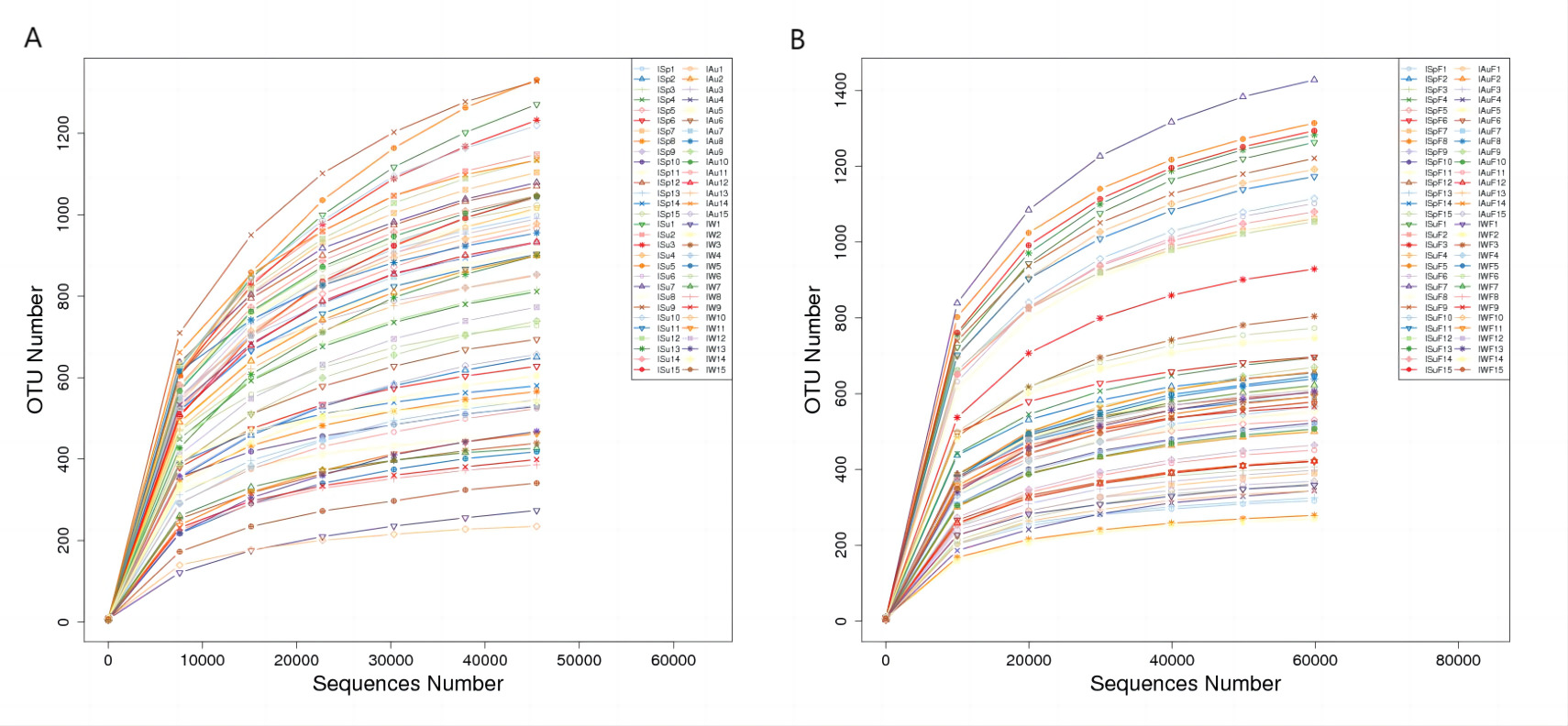

Supplement: SUPPLEMENTARY FIGURE S1 — (A) Rarefaction curve of oral group. (B) Rarefaction curve of gut group. When the curve tends to be flat, the amount of sequenced data is reasonable. [file Image_1.jpg]

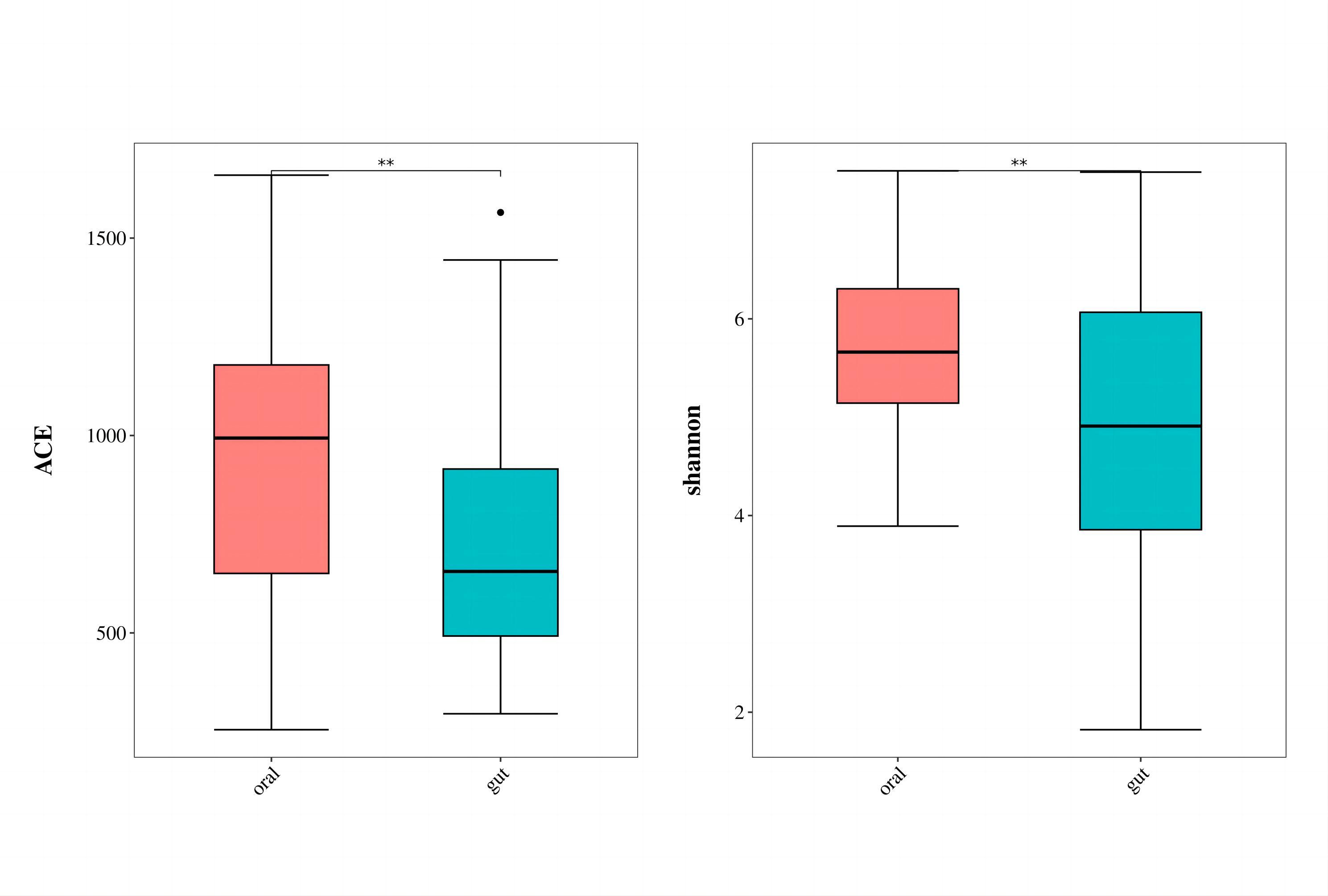

Supplement: SUPPLEMENTARY FIGURE S2 — (A) Comparison of the ACE index between the oral and gut groups. (B) Comparison of the Shannon index between the oral and gut groups. [file Image_2.jpg]

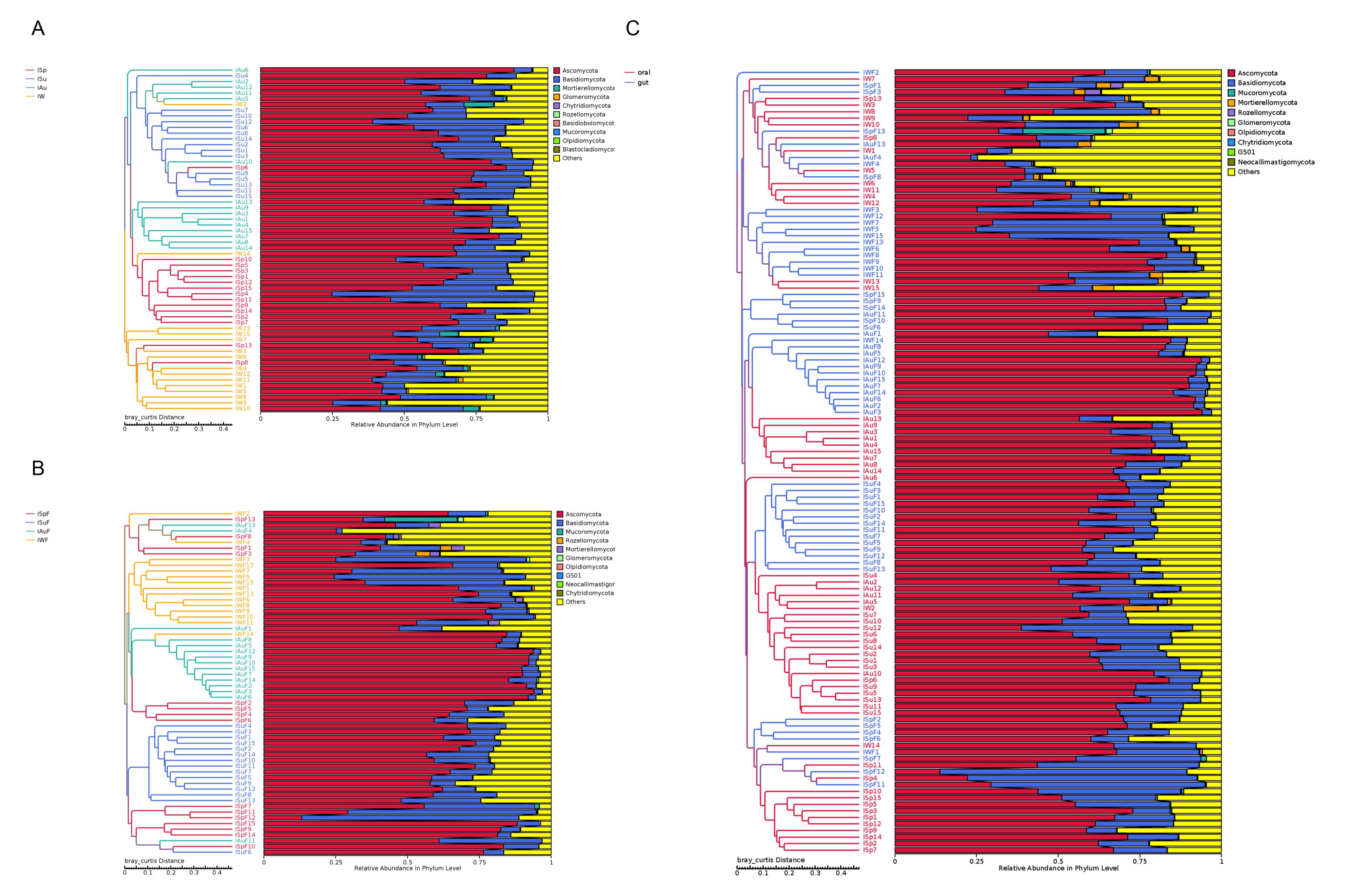

Supplement: SUPPLEMENTARY FIGURE S3 — UPGMA Clustering Tree Based on Bray Curtis. (A) Oral group, (B) gut group, (C) oral group vs gut group. [file Image_3.jpeg]

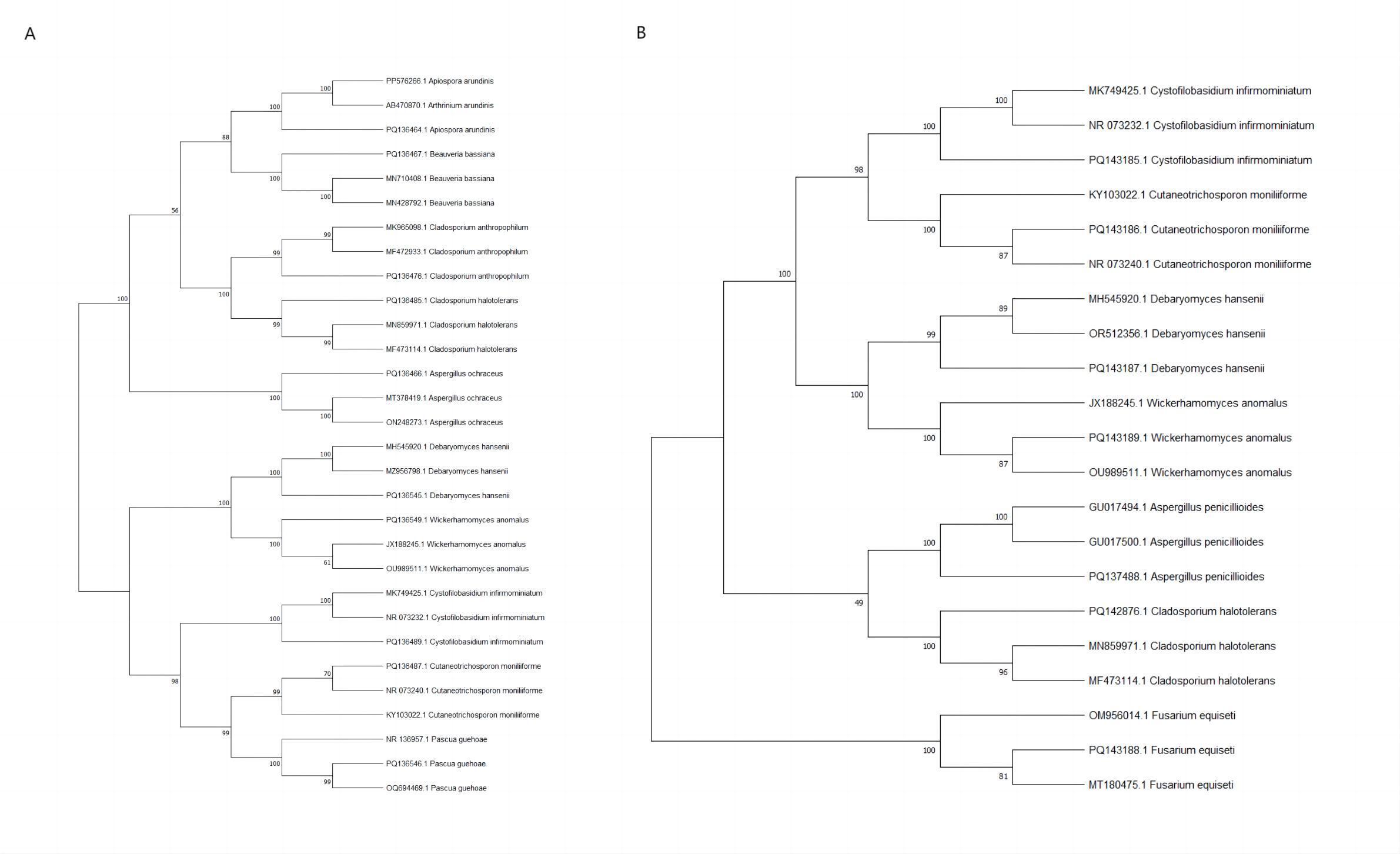

Supplement: SUPPLEMENTARY FIGURE S4 — Evolutionary relationships of taxa. The evolutionary history was inferred using the Neighbor-Joining method. (A) Oral group and (B) gut group. [file Image_4.jpg]

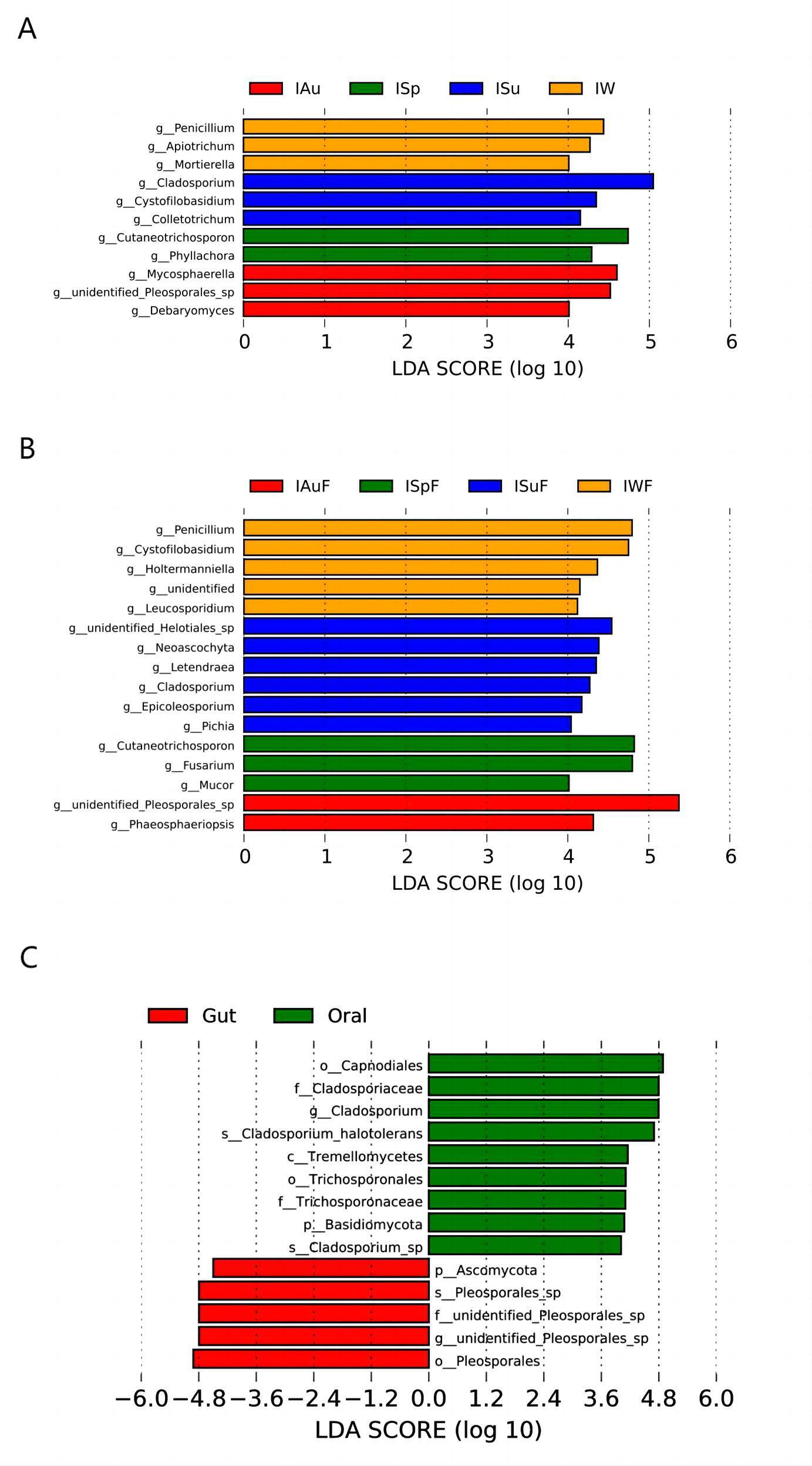

Supplement: SUPPLEMENTARY FIGURE S5 — LEfSe analysis of fungal communities (LDA>4, P<0.05). LEfSe analysis of oral (A) and gut (B) fungal communities across different seasons. (C) LEfSe analysis of fungal communities between the oral and gut environments. [file Image_5.jpg]

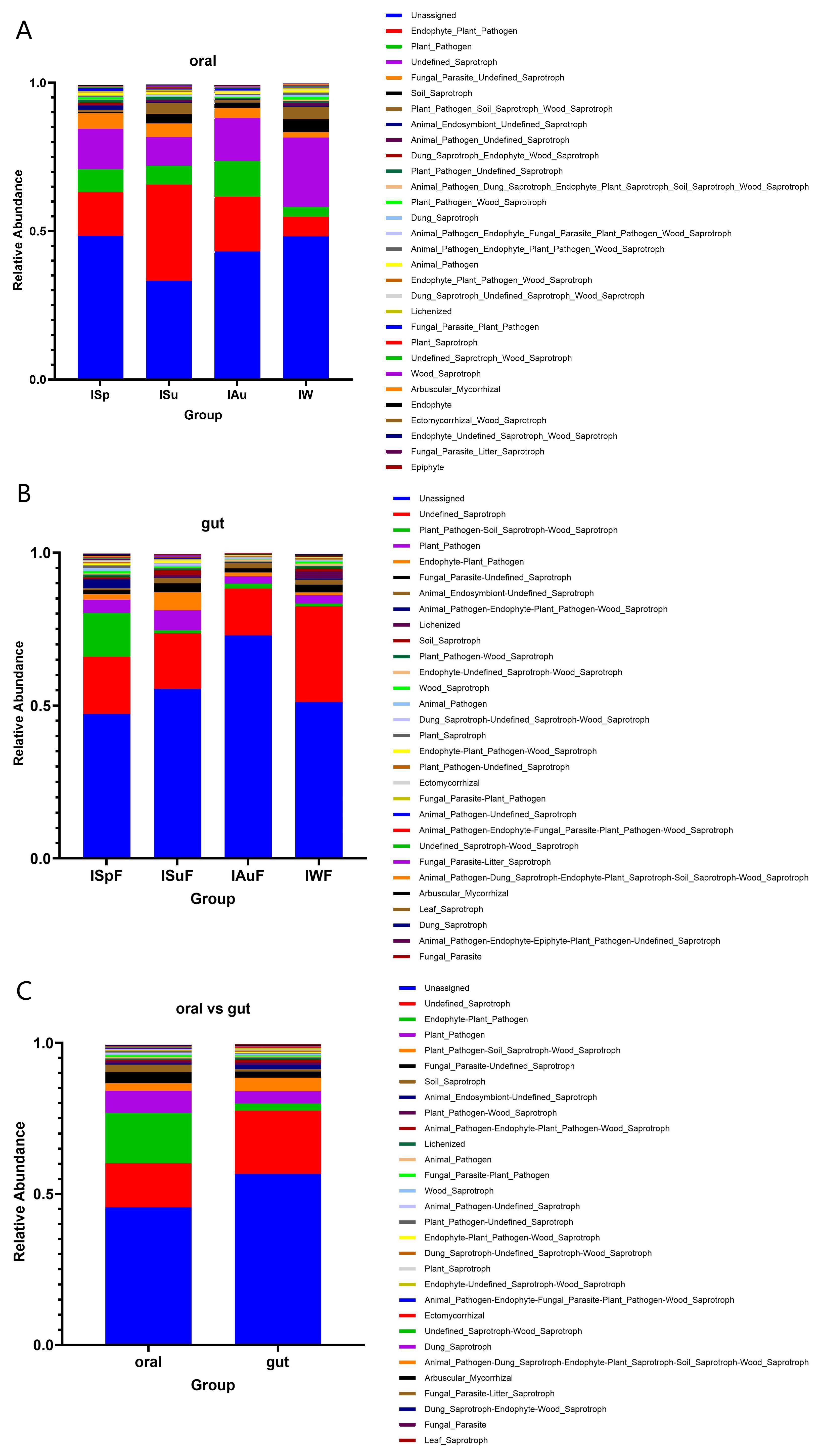

Supplement: SUPPLEMENTARY FIGURE S6 — Fungal functional category bar plot showing average percentage of FUNGuild functional annotation relative abundance in oral fungus. (A) Oral group across different seasons, (B) gut group across different seasons, and (C) oral group vs gut group. [file Image_6.jpg]
